# Monitoring of lysozyme thermal denaturation by volumetric measurements and nanoDSF technique in the presence of* N*-butylurea

**DOI:** 10.1007/s10867-019-09521-9

**Published:** 2019-03-22

**Authors:** Joanna Krakowiak, Magdalena Krajewska, Jarosław Wawer

**Affiliations:** Department of Physical Chemistry, Faculty of Chemistry, Gdańsk University of Technology, Narutowicza Str. 11/12, 80-233 Gdańsk, Poland

**Keywords:** Thermal denaturation, Protein stability, Specific volume, nanoDSF, Protein volume paradox, *N*-butylurea

## Abstract

The results of thermal studies of denaturation of hen egg white lysozyme (HEWL) in water and an aqueous solution of *N*-butylurea (BU) are presented. High-precision densimetric measurements were used to characterize and analyze the changes of the specific volume, *v*, during temperature elevation. The temperature of the midpoint of protein denaturation was also determined by nanoDSF technique (differential scanning fluorimetry). The densities of lysozyme solutions were measured at temperatures ranging from 298.15 to 353.15 K with an interval of 5 K at atmospheric pressure (0.1 MPa). The concentration of the protein covered the range from 2 to 20 mg per 1 ml of the solution. The optimal range of the concentration for the densimetric measurements was roughly estimated. In the transition region, the structural changes of the protein are accompanied by the biggest increase of *ν* values with temperature. Our measurements show that this effect can be monitored from volumetric data without precise determination of protein concentration. The results prove that a two-state model of denaturation could be used for data interpretation. Contrary to common misconception, the volumetric measurements suggest that the denatured protein does not necessarily need to be in a fully extended state. In this way, the ‘protein volume paradox’ could be explained. The surface area of the protein remains unchanged and thus the increase of the specific volume of the protein is relatively small. Additionally, the self-stabilizing effect of the protein in BU solution was reported. For the HEWL in pure water, this phenomenon was not observed.

**Electronic supplementary material:**

The online version of this article (10.1007/s10867-019-09521-9) contains supplementary material, which is available to authorized users.

## Introduction

The ordered structure of a protein can be relatively easily lost in the presence of chemical denaturants or due to increasing temperature. Thermal stability is commonly characterized by denaturation temperature *T*_*m*_, which is an important parameter describing the properties of proteins in a solution.

*T*_*m*_ depends on the internal structure of the macromolecule and the energetic profile of intramolecular bonds. It also provides indirect information about the interaction of the protein with the surrounding environment. The presence of a co-solute in a protein solution could change the stability of the macromolecule. In this situation, the change of the measured *T*_*m*_ would result from the alternation of the hydration layer around the protein or be the consequence of direct contact of the protein with the co-solute.

The main aims of this research are to determine prerequisites for successful measurements of *T*_*m*_ using densitometry. Most of all, we wish to specify the concentration range at which protein denaturation can be precisely observed. Apart from this, we define several, purely technical, requirements (e.g., importance of determination of the protein concentration, necessity of degassing the sample and the temperature range of the measurements).

To achieve this goal, the specific volumes of model protein hen egg white lysozyme (HEWL) were obtained in a temperature range of 25 to 80 °C. We performed the experiments in the presence of a protein destabilizer (*N*-butylurea) and in pure water. The results were compared with the data obtained from differential scanning fluorimetry (nanoDSF).

Similar research is often conducted using a variety of experimental techniques. Perhaps the simplest approach is to measure the intrinsic fluorescence of tryptophan [1]. In this method, the sample is excited by UV radiation at 280 nm. The recorded emission spectrum depends on the local conformation of the protein and the environment surrounding the fluorophore. During protein denaturation, the protein chain undergoes major structural rearrangements. The interactions of tryptophan and other fluorescent amino-acids (tyrosine, phenylalanine) with the surrounding residues are changing what, in turn, changes the recorded fluorescence signal.

Probably the most popular technique to study protein denaturation is differential scanning calorimetry (DSC) [2]. It allows observing the transition caused by the temperature increase. The recorded thermograms show the differences between the heat required to increase the temperature of the cell filled with the protein solution and the energy needed to increase the temperature of the cell filled with the buffer. This method allows, among others, to measure the denaturation temperature, enthalpy of the protein unfolding, and provides information about the mechanism of the denaturation.

In contrast to DSC, which provides a primarily thermodynamic description of the unfolding process, two other techniques, i.e., Fourier transform infrared spectroscopy (FT-IR) and circular dichroism (CD), are mainly focused on secondary structure transition. Usually, infrared spectroscopy is used to monitor the changes of the amide I (1600–1690 cm^−1^) and amide II bands (1480–1575 cm^−1^). The first of these regions is particularly sensitive to changes of the protein secondary structure [3]. In circular dichroism spectroscopy, the difference of the absorption of left- and right-handed circularly polarized light is measured. The spectra are recorded in the UV region from 260 nm to at least 190 nm. The obtained data can be used to determine the percentage of secondary structure components (α-helix, β-sheet, turns, unordered structures) for the protein molecule. This analysis can be easily performed using open-source algorithms [4, 5].

Volumetric measurements have a different character than the above-mentioned techniques. The strength of this method lies not only in its ability to detect the changes of the protein structure but also the alteration of the structure of the solvent that surrounds the macromolecule. Due to the sensitivity of densitometry to solvation-related phenomena, this technique is mainly used for the determination of the properties of hydration spheres of compounds at different temperatures [6, 7]. Unfortunately, the volumetric effects of transformations of proteins are less frequently studied [8, 9]. Volumetry could potentially supplement the results obtained from the other biophysical methods and provide a deeper insight into the studied phenomena.

It is known that dissolved low molecular weight substances can change the structure of water in many different ways [10]. It has been postulated that the properties of the protein will be different in such an altered environment [11]. In special cases, these effects can be perceived as water-mediated interactions between the co-solute and the protein. On the other hand, the changes of the protein conformation leading to the exposition of the different amino acids on its surface might be reflected by a change of the properties of the hydration sphere.

It must be emphasized here that the volumetric properties result from the total effect of the microscopic changes. Usually, this sum cannot be assigned to a particular region of the macromolecules or interpreted as a result of specific changes of intramolecular bonds. In this sense, the calculated volumes are macroscopic measures. Due to this fact, the experientially obtained volume of the solute, *v*, is often split into several contributions. In the simplified approach, it could be assumed that this parameter depends on the intrinsic volume of the solute and the volumetric effect caused by the solvent perturbation (solvation volume).

In this work, we divided the changes of the specific volume of the protein caused by temperature increase, Δ*v,* into three contributions [12]:

1 $$ \Delta v=\Delta {v}_{void}+\Delta {v}_{sol}+\Delta {v}_T $$

where Δ*v*_void_ is the change of the volume of voids inside the macromolecule, Δ*v*_sol_ is the change of the solvation volume and Δ*v*_T_ is the change of the thermal volume that is associated with the empty space around the protein. The justification of the above equation is given in ‘Supplementary Materials’ in this manuscript.

The solvation volume and the thermal volume depend on the surface area of the solute [13, 14]. As explained below, this observation is important for the interpretation of the results obtained in the present work.

The continuous increase of the temperature induces protein denaturation. After reaching the critical value of the temperature, the monotonic trend of *v* = *f*(*T*) overlaps with the abrupt change caused by the protein denaturation, Δ*v*_U_. The van der Waals volumes of residues constituting the molecule do not change during this transition but the internal voids are significantly reduced [12, 15]. The increase and the changes on the surface of the protein change the solvation related phenomena [16]. It was also postulated that changes of the thermal term contribute significantly to volumetric effects caused by the protein denaturation [17, 18].

To interpret the changes of the specific volume, it is necessary to define the model of the denaturation process. In a crude approximation, the peptide chain unfolds and some (or all) residues of amino acids are exposed to the solvent [15, 18]. Non-polar amino acids are transferred from the interior of the macromolecule to its surface and the secondary structure of the protein is mostly lost [19].

This simple model leads to the so-called ‘protein volume paradox’ [18, 20]. The solvation of the polar and the charged groups gives a negative contribution to the volume. The volume of transfer of the non-polar compounds from the non-polar environment to the water is also negative. The internal voids become accessible to the solvent thus upon denaturation Δ*v*_void_ < 0. Consequently, it could be expected that the protein unfolding should result in a large and negative volumetric effect. Opposite to that, the recorded changes of the proteins specific volumes caused by denaturation are small and with a positive or negative value [9].

The discrepancies between expected and measured values could be explained in different ways [20, 21]. The large negative effects could be compensated by the significant positive contribution. It has been suggested that the big increase in the thermal volume Δ*v*_T_ could be the missing factor not taken into consideration [18]. It is also possible that the hydration of the non-polar residues gives a positive contribution to the change of the volume during protein unfolding [10]. It was also questioned if the transfer studies of the non-polar compounds provide a good reflection of the protein unfolding. A more precise estimation of the volumes of the residues buried in the core of protein [19] shows that the cancellation of the volumetric effects for polar and non-polar residues could be responsible for the small value of Δ*v*_U_. According to the alternative explanation, the denatured protein is not fully extended to the solution and its interior remains to a significant degree inaccessible to the solvent [13].

The analysis described herein is based on high-precision density measurements of the solutions of the protein and the solvent used for the sample preparation. The denaturation studies were performed for the model protein: lysozyme from chicken egg white (HEWL). Two sets of experiments were conducted, i.e., the protein was dissolved in pure water or in an aqueous solution of* N*-butylurea (0.5 M).

Pure water was used in the first experiment to keep the systems in study as simple as possible. This approach makes the interpretation easier and the obtained results can be related to other works for which the number of the components in the solution must be minimized.

*N*-butylurea (BU) was chosen for the research due to the unusual properties of the hydration sphere formed around this compound [10]. Despite its non-polar character, BU does not break the structure of the water but makes it more reinforced. This observation suggests that the presence of BU would affect significantly the properties of the HEWL.

The specific volume of HEWL, *v*, was measured from 25 to 80 °C. From the collected data, the denaturation temperature of this protein was estimated. These results were compared with the values obtained from the nanoDSF measurements. It has been demonstrated that thermal denaturation causes marked volume changes and densimetric measurements allow monitoring denaturation of the protein provided that the protein concentration is sufficiently high. The denaturation temperature can be determined even if the precise concentration of the protein is not known.

## Materials and methods

Hen egg white lysozyme (HEWL, Fluka, Cat. No. 62971) was dialyzed against deionized water and lyophilized.* N*-butylurea (BU, Fluka, Cat. No. 19940, purity ≥ 0.99) was used as received. The solutions were prepared by weight using analytical balance (Radwag WAA 40/160/X/1, Poland) with a precision of 0.1 mg. The working solutions were prepared by dissolution of the lysozyme in pure water or in* N*-butylurea aqueous solution (0.5 M) and filtrated four times by a 0.1-μm syringe filter. Deionized water for solution preparation was degassed by boiling for approximately 20 min. The samples were additionally degassed under reduced pressure just before density measurements. The concentration of the protein determined by weight was equal to 2, 5, 10, or 20 mg per 1 ml of water or aqueous solution of* N*-butylurea.

The exact protein concentration was determined spectroscopically. UV-Vis absorbance spectra were recorded on a Thermo Evolution 300 spectrophotometer. Lysozyme concentration was calculated from absorbance at 280 nm using the extinction coefficient ε = 2.65 dm^3^·(g·cm)^−1^. Three scans were averaged for each sample. Each sample was examined by two techniques: high-precision densimetry and native differential scanning fluorimetry.

Densities of the liquids were measured using Anton Paar DMA 5000 densimeter with a precision of 1.0·10^−3^ kg·m^−3^. The temperatures ranged from 298.15 K up to 353.15 K with an interval of 5 K. The instrument was equipped with a Peltier-type thermostating unit and the temperature was kept constant to within ± 0.001 K of the atmospheric pressure.

The uncertainty of the determination of the step increment of the specific volume of lysozyme was estimated using the total differential method according to the equation:

2 $$ \Delta y=2\cdotp \Sigma \mid \partial f\left({x}_i\right)/\partial {x}_i\mid \cdotp \Delta {x}_i $$

where *f*(*x*_*i*_) is the relation used to calculate the specific volume of the protein (Eq. 3) and Δ*x*_*i*_ are uncertainties of the variables from Eq. (3) for a given concentration of the protein.

The thermal denaturation curves were determined by measurements of protein intrinsic fluorescence. This analysis was performed using label-free, native differential scanning fluorimetry (nanoDSF; apparatus: Prometheus NT.48, NanoTemper). The tryptophan residues of the protein were excited at 280 nm and the fluorescence intensity was recorded at 330 and 350 nm. The temperature of the measurement compartment increased from 25 to 95 °C at a rate of 2 deg.·min^−1^.

## Results and discussion

The specific volume of lysozyme, *v* [cm^3^·g^−1^], at each temperature was calculated from the equation:

3 $$ v=\frac{1}{d_0}-\frac{d-{d}_0}{d_0\cdot c} $$

where *d* and *d*_0_ [g·cm^−3^] are the densities of the protein solution and solvent (i.e., water or an aqueous solution of BU), respectively; *c* [g·cm^−3^] is lysozyme concentration. The densities and the calculated values of the specific volumes are collected in Tables S1-S2 in the Supplementary Materials.

**Fig. 1:**
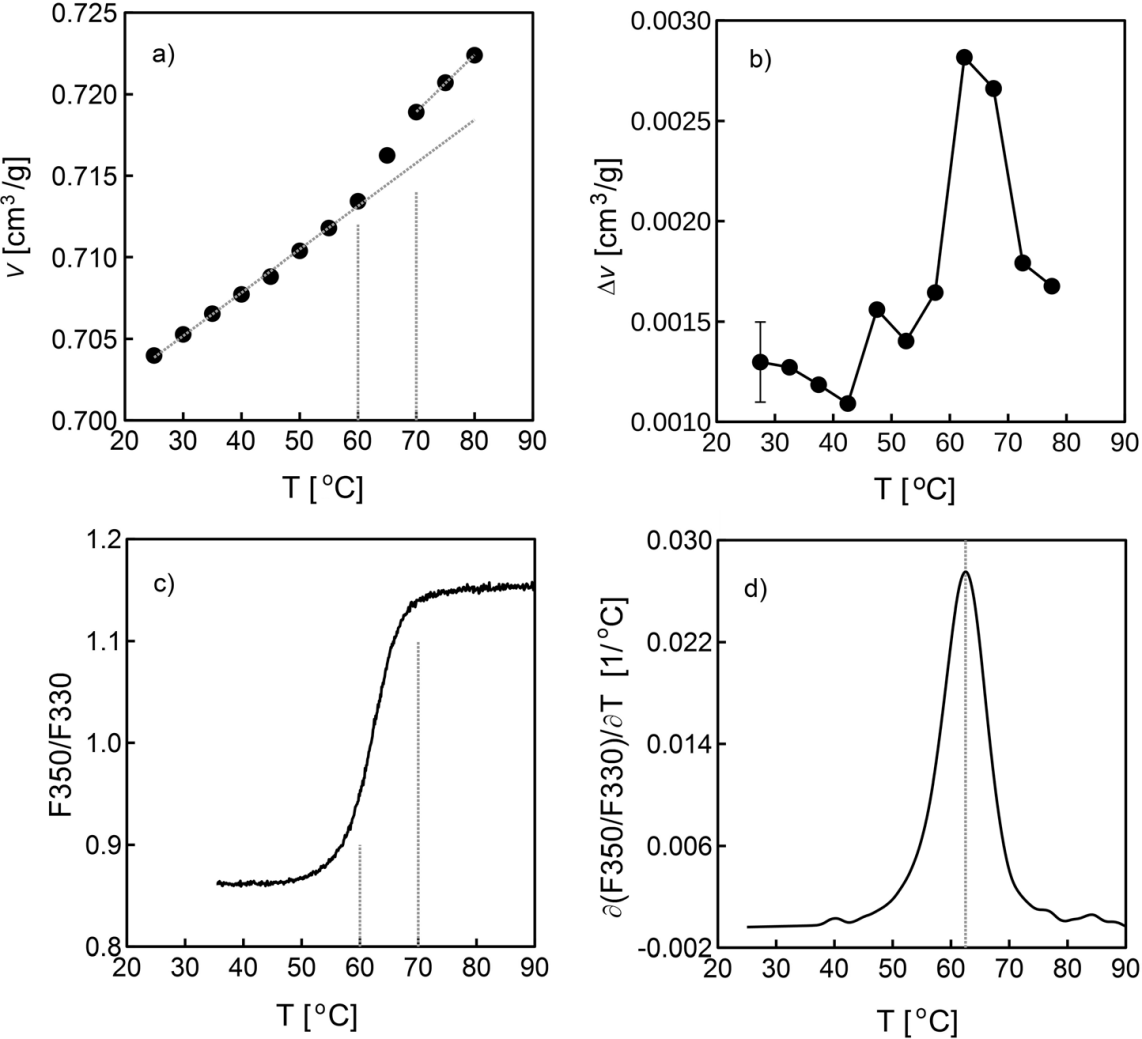
Thermal denaturation of lysozyme in aqueous solution containing 20 mg/ml of lysozyme and 0.5 M* N*-butylurea. The influence of the temperature on: **a** the specific volume of lysozyme, *v*; **b** the change of the specific volume, Δ*v*, of lysozyme Δ*v* = *v*(T_2_) - *v*(T_1_), temperature points taken as an average of T_2_ and T_1_; **c** the ratio of the intensity of fluorescence at 350 and 330 nm, F350/F330; and** d** the temperature derivative of (F350/F330) data

The representative results collected during the thermal denaturation of HEWL are presented in Fig. 1a–d. These data were recorded for the samples containing 20 mg/ml of lysozyme in a 0.5 M aqueous solution of* N*-butylurea. The results obtained for the other samples are analogous.

To monitor the denaturation process of HEWL, by the use of the densimetric measurements, the specific volume of the protein calculated according to Eq. (3) was plotted as a function of temperature (Fig. 1a). As can be seen, *v* of the protein increases as the temperature increases. The discussed *v* = *f*(*T*) relation can be divided into three distinctively different parts. Each of them can be ascribed to different processes occurring during the temperature elevation. In the first phase, the linear increase of *v* with the temperature may be associated with the change of the volume of the native state of lysozyme due to thermal expansion of the protein and the increase of the volume of the hydration sphere. In the second part of the plot (from 60 to 70 °C), the increase of the specific volume with temperature is much bigger and it is related to the denaturation of the protein. The last third part results from the thermal expansion of the denatured protein. Interestingly, it appears that the slope of this section is similar to the slope of the first part of the plot obtained for the native protein.

**Fig. 2:**
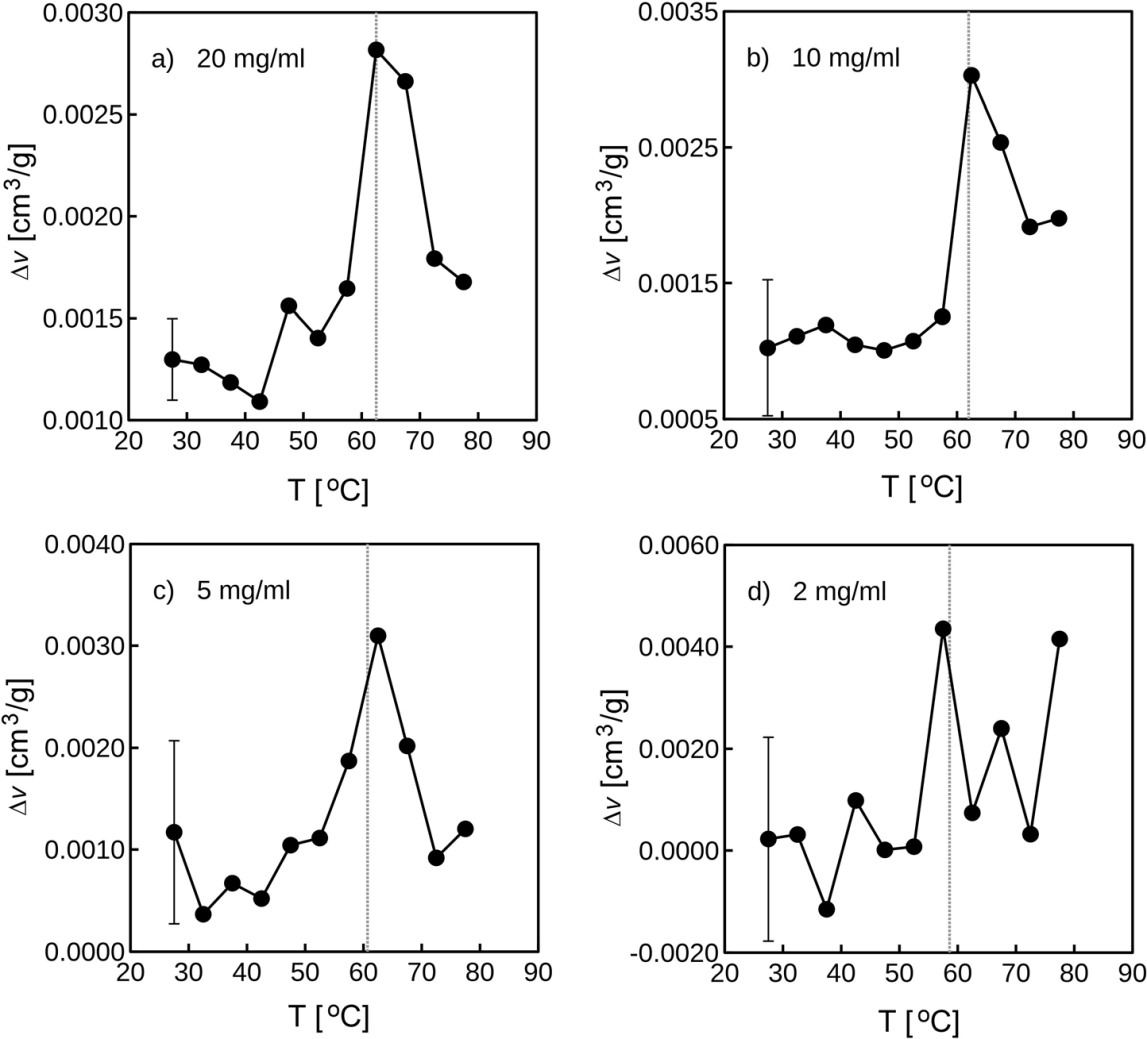
The change of the specific volume, Δ*v*, of lysozyme against the temperature; Δ*v* = *v*(T_2_) - *v*(T_1_), temperature points taken as an average of T_2_ and T_1_. The concentration of lysozyme [mg/ml]: **a** 20; **b** 10; **c** 5; **d** 2 in 0.5 M aqueous solution of* N*-butylurea. The* vertical dotted line* shows the denaturation temperature of lysozyme obtained using nanoDSF technique

**Fig. 3:**
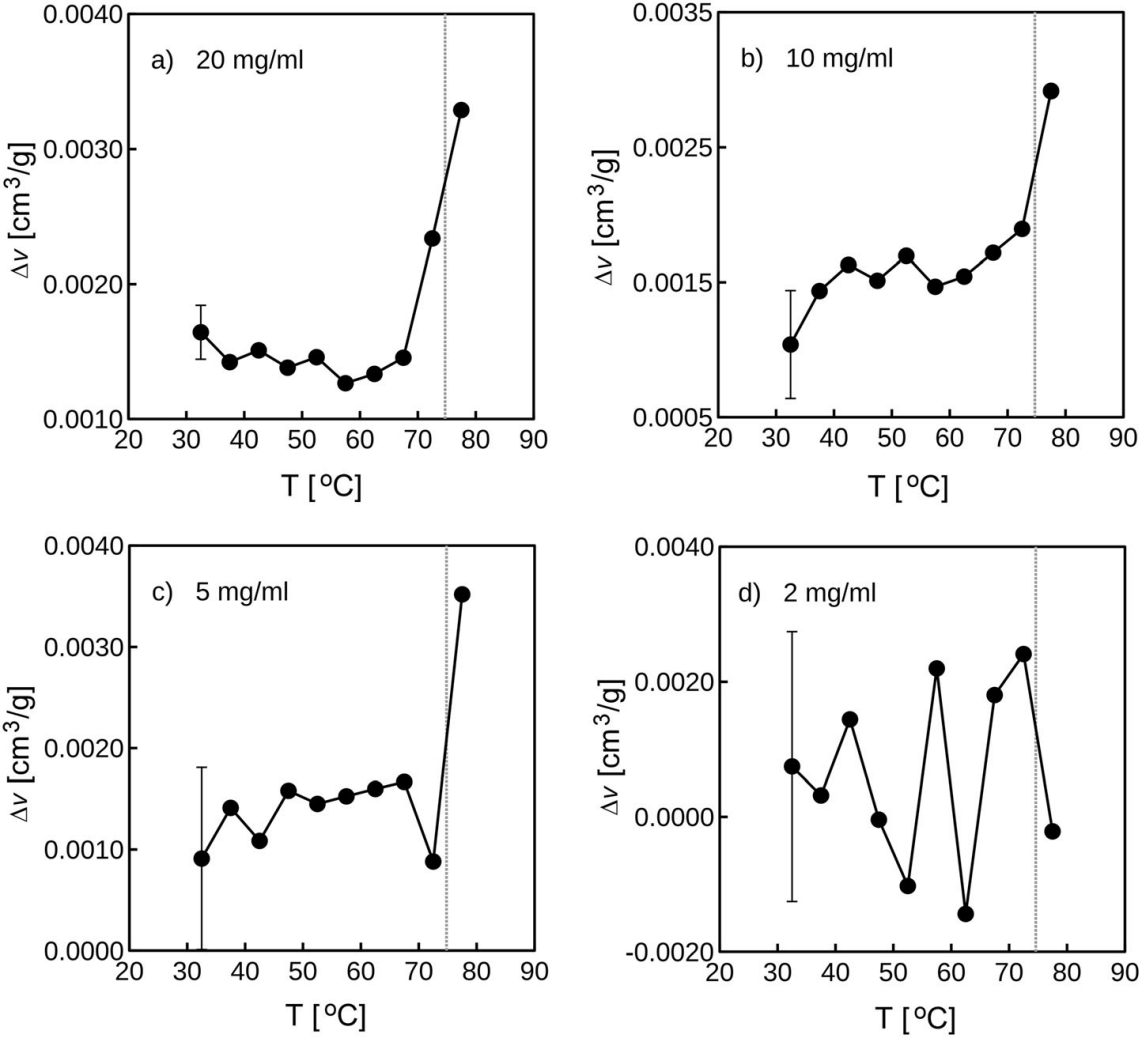
The change of the specific volume, Δ*v*, of lysozyme against the temperature; Δ*v* = *v*(T_2_) - *v*(T_1_), temperature points taken as an average of T_2_ and T_1_. The concentration of lysozyme [mg/ml]: **a** 20; **b** 10; **c** 5; **d** 2 in water. The* vertical dotted line* shows the denaturation temperature of lysozyme obtained using nanoDSF technique

The obtained *v* = *f*(*T*) relations are smooth and devoid of significant measurement error. The presence of distinct phases of the denaturation becomes apparent after plotting the step increment of the specific volume Δ*v* against temperature (Figs. 1b, 2, and 3). The resulting curves Δ*v* = *f*(*T*) are far noisier because the absolute values of Δ*v* are small. However, the ongoing protein denaturation can be easily detected.

In the case of the HEWL solutions in pure water (Fig. 3a–d), only the two first stages are observed i.e., linear increase of the specific volume of the native lysozyme and transition of the HEWL to the partially melted state. The complete experimental curves on which all three states of protein are visible (native, partially melted, denatured) were recorded for HEWL in* N*-butylurea solution (Fig. 2a–d). It was possible to obtain these results due to the much lower denaturation temperature of the lysozyme in the presence of BU than the maximal temperature of the densimetric measurements (80 °C).

The midpoint denaturation temperature, *T*_*m*_, of the lysozyme is expected within the temperature range where the highest change of *v* of the lysozyme is observed. In other words, it should be in accordance with the highest reported value of Δ*v*. To achieve better precision of the determination of *T*_*m*_, it would be necessary to decrease the step of the temperature increment interval.

Apart from the volumetric studies, the thermal denaturation of lysozyme was monitored by the nanoDSF technique (differential scanning fluorimetry). This method allowed an unambiguous determination of the protein transition midpoint temperature, *Tm* and helped to interpret the plots of the temperature dependency of the specific volume of lysozyme. In this technique, the temperature of the sample is increased and the fluorescence of the protein tryptophans is measured [22, 23].

Figure 1c presents the ratio of the intensities of fluorescence measured at 350 and 330 nm (F350/F330) as a function of temperature obtained for a solution of HEWL (20 mg/ml) in the aqueous solution of* N*-butylurea (0.5 M). The denaturation temperature of the protein corresponds to the temperature where the biggest increase of the fluorescence is observed (the inflection point of the plot). This value is obtained from the temperature derivative of the (F350/F330) = *f*(*T*) relation and is equal to 62.5 °C for this system (Fig. 1d). The *T*_*m*_ value determined from the nanoDSF measurements is in excellent agreement with the denaturation temperature estimated based on density measurements. Moreover, as can be seen in Fig. 1a, c, the change of the *v* data with temperature correlates with the change of the spectra signal, which indicates that both of these techniques are sensitive to the same transition effects in the lysozyme.

The accuracy of the obtained *v* values depends on the accuracy of the density measurements and the accuracy of the determination of the concentration of the protein. However, when the changes of the specific volumes Δ*v* are analyzed, the exact value of the protein concentration does not need to be known. The Δ*v* values obtained for approximated values of concentrations of the protein (calculated from weights) and those based on more accurate values calculated from the spectroscopic data are virtually identical. The differences between the two sets are much smaller than the error of the method.

The precision of Δ*v* data depends mainly on the differences between densities of the solution and the solvent and is related to the accuracy of the density measurements. The bigger value of (*d* - *d*_0_) gives the smaller error of Δ*v* and in this way a more accurate description of the studied systems is achieved. The (*d* - *d*_0_) value is associated with the amount of the solute introduced to the pure solvent. In this work, among other problems that we wished to address, we have focused on the estimation of the optimal lysozyme concentration needed for study of thermal stability of the protein by density measurements.

In order to pursue this goal, we performed volumetric experiments for four different concentrations of lysozyme (2, 5, 10, and 20 mg/ml). As can be seen in Figs. 2 and 3, the obtained temperature relations depend on protein concentration for both sets of solutions in pure water and in the presence of BU. In the case of the smallest amount of lysozyme (2 mg/ml) dissolved in the solutions, the difference between solution and solvent densities is small compared to the accuracy of density measurements. The obtained Δ*v* data are noisy and seriously biased by measurement error. The proper interpretation of the plots presented in Figs. 2d and 3d is impossible.

The relations Δ*v* = *f*(*T*) for the highest concentrations of lysozyme are similar to each other. The processes mentioned above occurring during lysozyme denaturation can be clearly observed and the estimation of the denaturation temperature is an easy task.

The vertical lines placed in Figs. 2a–d and 3a–d denote the denaturation temperature obtained from differential scanning fluorimetry. The obtained *T*_*m*_ values from both techniques are listed in Table 1. As can be seen, the agreement of the *T*_*m*_ data between two experimental methods is very good. Of course, better accordance is noted for BU solutions due to a significantly lower value of denaturation temperature.

**Table 1:** Denaturation temperature, *T*_*m*_, of lysozyme in the absence and in the presence of co-solute (*N*-butylurea) determined by authors and taken from literature

Solvent	Technique	Lysozyme concentration [mg/ml]			
		2	5	10	20
		Denaturation temperature *T*_*m*_ [°C]			
Water	NanoDSF	74.6	74.8	74.7	74.7
	Densitometry	> 70	> 70	> 70	> 70
0.5 M *N*-butylurea	NanoDSF	58.6	60.7	62.0	62.5
	Densitometry	≈ 57.5	62.5	62.5	62.5
Water (literature data)		74.5^a^; 75.2^b^; 77.19^c^; 77.4^d^; 77.5^e^			

^a^[24], ^b^[25], ^c^[26], ^d^[27], ^e^[28]

The nanoDSF technique revealed that the denaturation temperature of lysozyme in solution containing BU depends on the protein concentration. The *T*_*m*_ values for the solutions containing 2 and 5 mg/ml of HEWL are markedly lower than *T*_*m*_ values obtained for the concentrations 10 and 20 (which are very close to each other). This means that thermal stability increases as the concentration of the protein increases. The properties of the solvation spheres formed around solutes exert a tremendous impact on the protein behavior. The co-solutes with the hydration layer similar to the solvation layer of the protein act as structure stabilizers and increase *T*_*m*_ [11]*.* The increase of the concentration of the protein is equivalent to the addition of the similarly hydrated co-solute; as a result, the self-stabilizing effect is present. The above-mentioned effect was not observed for the solution of HEWL in pure water.

As we mentioned earlier, the temperature increment of a specific volume of HEWL after denaturation is comparable to Δ*v* for the native state of lysozyme. A significant increase of volume has been reported only for transformation of the native protein to the denatured globule. This simple observation has a great significance. Firstly, it supports the common assumption that denaturation could be regarded as a two-state phenomenon [9]. Secondly, it provides information regarding the structure of the denatured protein.

The relative small increase of the volume of the lysozyme during denaturation Δ*v*_U_ and the comparable values of the Δ*v* for native and denatured HEWL (Fig. 2a–c) imply that the denaturation of the protein is not accompanied by the extensive change of the exposition of the amino-acids to the solution. This observation contradicts the hypothesis that the protein in the denatured state must be in an extended state [15, 18]. The increased exposition of the amino-acids to the solvent would result in a considerable increase in the solvent accessible surface area of the macromolecule and a significant change of the hydration phenomena. Both of these factors would result in large changes of the protein volume Δ*v*_U_ and most importantly the dissimilar values of the temperature increment of the specific volume of HEWL before and after denaturation.

The popular term ‘unfolded molecule’ used as a synonym of the denatured state could be misleading because it implies directly that the amino acids from the interior of the protein are exposed to the solvent. Our findings are in agreement with the literature data for different systems [12, 13, 16].

However, the reader must keep in mind that every system in study needs to be analyzed separately. The reported changes of the volume with the temperature could be similar to our results [9, 17] but also distinctively different [8, 29]. Moreover, the volumetric effects depend largely on the denaturation conditions [12].

## Conclusions

Reliable parameters that describe protein denaturation can be obtained from volumetric measurements on the conditions that: (i) the denaturation temperature is much lower (at least 15°) than the highest temperature of a measured density; (ii) the protein concentration is sufficiently high (above 10 mg/ml) but the determination of the denaturation temperature does not require precise determination of the protein concentration; (iii) the temperature interval is in accordance with the required precision of the study (iv) the liquid samples are carefully degassed just before density measurements.

Our densimetric investigations suggest that HEWL denaturation can be regarded as a two-state process. However, contrary to widespread belief, there is no extensive change of the exposition of the amino-acids on the surface of the protein during denaturation.

The volumetric measurements provide insight into the thermal denaturation of the protein and can supplement other biophysical methods. The obtained results could be potentially important for both fundamental research and applied science.

## Electronic supplementary material

ESM 1 (DOC 126 kb)
