# Supplementary material for: Monitoring of lysozyme thermal denaturation by volumetric measurements and nanoDSF technique in the presence of N-butylurea
Source: J Biol Phys. 2019 Mar 22;45(2):161–72. doi: 10.1007/s10867-019-09521-9 (PMC6548760; doi:10.1007/s10867-019-09521-9)
Supplement: Supplementary file 1 — (DOC 126 kb) [file 10867_2019_9521_MOESM1_ESM.doc]

**Specific volume of the protein - volume contributions**

In a first approximation the specific volume of the protein, *v*, could be divided into three contributions: the intrinsic volume of the solute, *v_int_*, the changes of the volume of the solvent caused by the perturbation of its structure by the solute, *v_sol_*, and the kinetic contribution to the specific volume *β_TO_RT* [1]⁠.

*v* = *v_int_* + *v_sol_* + *β_TO_RT* (1)

More precise descriptions [2–4] include other, sometimes non-orthogonal, factors. The intrinsic volume (cavity volume), is the part of the solution occupied by the protein and thus it is not penetrated by solvent. The *v_int_* consists of the Van der Waals volume of the atoms of the dissolved protein *v_VdW_* and the volume of voids inside the macromolecule, *v_void_*. The *v_int_* also includes thermal volume, *v_T_*, which could be regarded as an empty space around protein:

*v_int_* = *v_VdW_* + *v_void_* + *v_T_* (2)

The *v_T_*, could be split into three components: steric, vibrational and structural. Steric component describes imperfect packing of the solute in the solution (the solute and solvent molecules are not cubical objects), vibrational component results from the thermal oscillations of the solute and the atoms constituting the solvent molecules, the structural component results from the tetrahedral structure of water molecules:

*v_T_* = *v_steric_* + *v_vibr_* + *v_structural_* (3)

The perturbation volume, or more correctly the solvation volume *v_sol_* (eq. 1) reflects the change of the volume of the solvent due to the presence of the solute-solvent interactions and the rearrangement of the solvent-solvent interactions. The *v_sol_* depends on the number of the water molecules affected by the solute and the difference between molar volume of water in the solvation shell and the molar volume of bulk water. The discussed interactions are different for charged residues for which the electrostriction dominates and for polar groups which are able to form hydrogen bonds and the hydrophobically hydrated non-polar residues [5, 6].⁠

The ideal term in the equation 1 describing the kinetic contribution to the specific volume i.e. the *β_TO_RT* is also sometimes discussed. The *β_TO_* denotes the isothermal compressibility of the solvent, *R* and *T* are gas constant and the temperature, respectively. The absolute value of this term is small in the comparison to the total volume of macromolecule and can be omitted [4]⁠.

The specific volume of the protein depends greatly on the intrinsic volume but the *v* value is also sensitive, to a certain degree, to the hydration related effects [4]⁠. The solvation volume and the thermal volume depend on the surface area of the solute [2, 4].

The temperature increase leads to the change of the specific volume of the protein Δ*v*. Similarly to the *v*, the Δ*v* can be divided into contributions and then their thermal response could be analysed.

The Van der Waals volume of the solute does not depend significantly on the temperature. The volume changes Δ*v* result from the: alterations of the internal void volume Δ*v_void_*, the changes of properties of water in the solvation sphere with respect to the changes of the bulk water Δ*v_sol_* and from the changes of the thermal volume Δ*v_T_* [5]⁠:

Δ*v* = Δ*v_void_* + Δ*v_sol_* + Δ*v_T_* (4)

**References**

1. Murphy, L.R., Matubayasi, N., Payne, V.A., Levy, R.M.: Protein hydration and unfolding – insights from experimental partial specific volumes and unfolded protein models. Fold. Des. 3, 105–118 (1998)

2. Lee, S., Tikhomirova, A., Shalvardjian, N., Chalikian, T. V.: Partial molar volumes and adiabatic compressibilities of unfolded protein states. Biophys. Chem. 134, 185–199 (2008)

3. Schweiker, K.L., Fitz, V.W., Makhatadze, G.I.: Universal Convergence of the Specific Volume Changes of Globular Proteins upon Unfolding. Biochemistry. 48, 10846–10851 (2009)

4. Chalikian, T. V., Totrov, M., Abagyan, R., Breslauer, K.J.: The Hydration of Globular Proteins as Derived from Volume and Compressibility Measurements: Cross Correlating Thermodynamic and Structural Data. J. Mol. Biol. 260, 588–603 (1996)

5. Sirotkin, V.A., Winter, R.: Volume Changes Associated with Guanidine Hydrochloride, Temperature, and Ethanol Induced Unfolding of Lysozyme. J. Phys. Chem. B. 114, 16881–16886 (2010)

6. Wawer, J., Krakowiak, J.: Structural changes of water caused by non-electrolytes: Volumetric and compressibility approach for urea-like analogues. J. Mol. Liq. 259, 112–123 (2018)

**Table S. 1.**

The densities of the solutions, *d*, and solvent, *d_0_*, measured at temperatures (303.15-353.15) K for aqueous solutions of lysozyme of concentration *c* in pure water.

| *T* / [K] | *c*/[mg/ml] | *d_0_* / [g/ml] | *d* / [g/ml] | *v* / [ml/g] |
| --- | --- | --- | --- | --- |
| 303.15 | 2  (1.95)^a^ | 0.995672 | 0.996203 | 0.7305 |
| 308.15 |  | 0.994050 | 0.994581 | 0.7312 |
| 313.15 |  | 0.992230 | 0.992762 | 0.7315 |
| 318.15 |  | 0.990225 | 0.990756 | 0.7330 |
| 323.15 |  | 0.988045 | 0.988578 | 0.7329 |
| 328.15 |  | 0.985699 | 0.986236 | 0.7319 |
| 333.15 |  | 0.983200 | 0.983735 | 0.7341 |
| 338.15 |  | 0.980550 | 0.981090 | 0.7327 |
| 343.15 |  | 0.977762 | 0.978301 | 0.7345 |
| 348.15 |  | 0.974838 | 0.975375 | 0.7369 |
| 353.15 |  | 0.971778 | 0.972318 | 0.7367 |
| 303.15 | 5  (4.91)^a^ | 0.995652 | 0.997046 | 0.7185 |
| 308.15 |  | 0.994033 | 0.995426 | 0.7194 |
| 313.15 |  | 0.992215 | 0.993605 | 0.7208 |
| 318.15 |  | 0.990210 | 0.991599 | 0.7219 |
| 323.15 |  | 0.988031 | 0.989417 | 0.7235 |
| 328.15 |  | 0.985689 | 0.987073 | 0.7249 |
| 333.15 |  | 0.983191 | 0.984573 | 0.7264 |
| 338.15 |  | 0.980543 | 0.981923 | 0.7280 |
| 343.15 |  | 0.977756 | 0.979134 | 0.7297 |
| 348.15 |  | 0.974833 | 0.976213 | 0.7306 |
| 353.15 |  | 0.971778 | 0.973148 | 0.7341 |
| 303.15 | 10  (9.98)^a^ | 0.995651 | 0.998394 | 0.7278 |
| 308.15 |  | 0.994030 | 0.996770 | 0.7288 |
| 313.15 |  | 0.992212 | 0.994946 | 0.7303 |
| 318.15 |  | 0.990208 | 0.992935 | 0.7319 |
| 323.15 |  | 0.988029 | 0.990751 | 0.7334 |
| 328.15 |  | 0.985687 | 0.988403 | 0.7351 |
| 333.15 |  | 0.983189 | 0.985902 | 0.7366 |
| 338.15 |  | 0.980541 | 0.983251 | 0.7381 |
| 343.15 |  | 0.977753 | 0.980459 | 0.7398 |
| 348.15 |  | 0.974829 | 0.977530 | 0.7417 |
| 353.15 |  | 0.971774 | 0.974461 | 0.7446 |
| 303.15 | 20  (19.1)^a^ | 0.995648 | 1.001124 | 0.7165 |
| 308.15 |  | 0.994031 | 0.999489 | 0.7182 |
| 313.15 |  | 0.992214 | 0.997660 | 0.7196 |
| 318.15 |  | 0.990210 | 0.995644 | 0.7211 |
| 323.15 |  | 0.988031 | 0.993457 | 0.7225 |
| 328.15 |  | 0.985687 | 0.991105 | 0.7239 |
| 333.15 |  | 0.983187 | 0.988602 | 0.7252 |
| 338.15 |  | 0.980539 | 0.985951 | 0.7265 |
| 343.15 |  | 0.977751 | 0.983159 | 0.7280 |
| 348.15 |  | 0.974829 | 0.980218 | 0.7303 |
| 353.15 |  | 0.971775 | 0.977129 | 0.7336 |

^a^ concentration value determined spectroscopically taken as mass fraction [mg_protein_/g_solution_]

**Table S. 2.**

The densities of the solutions, *d*, and solvent, *d_0_*, measured at temperatures (303.15-353.15) K for aqueous solutions of lysozyme of concentration *c* in the presence of 0.5 M n-butylurea.

| *T* / [K] | *c*/[mg/ml] | *d_0_* / [g/ml] | *d* / [g/ml] | *v* / [ml/g] |
| --- | --- | --- | --- | --- |
| 303.15 | 2  (1.95)^a^ | 0.998555 | 0.999096 | 0.7235 |
| 308.15 |  | 0.996698 | 0.997240 | 0.7238 |
| 313.15 |  | 0.994651 | 0.995197 | 0.7227 |
| 318.15 |  | 0.992431 | 0.992977 | 0.7236 |
| 323.15 |  | 0.990047 | 0.990595 | 0.7237 |
| 328.15 |  | 0.987504 | 0.988054 | 0.7237 |
| 333.15 |  | 0.984818 | 0.985362 | 0.7281 |
| 338.15 |  | 0.981986 | 0.982531 | 0.7288 |
| 343.15 |  | 0.979023 | 0.979566 | 0.7312 |
| 348.15 |  | 0.975931 | 0.976476 | 0.7315 |
| 353.15 |  | 0.972719 | 0.973259 | 0.7357 |
| 303.15 | 5  (4.47)^a^ | 0.998552 | 0.999813 | 0.7189 |
| 308.15 |  | 0.996693 | 0.997956 | 0.7193 |
| 313.15 |  | 0.994647 | 0.995911 | 0.7199 |
| 318.15 |  | 0.992427 | 0.993693 | 0.7204 |
| 323.15 |  | 0.990042 | 0.991308 | 0.7215 |
| 328.15 |  | 0.987502 | 0.988768 | 0.7226 |
| 333.15 |  | 0.984811 | 0.986074 | 0.7245 |
| 338.15 |  | 0.981983 | 0.983238 | 0.7276 |
| 343.15 |  | 0.979019 | 0.980271 | 0.7296 |
| 348.15 |  | 0.975925 | 0.977179 | 0.7305 |
| 353.15 |  | 0.972712 | 0.973967 | 0.7317 |
| 303.15 | 10  (9.03)^a^ | 0.998476 | 1.001112 | 0.7094 |
| 308.15 |  | 0.996619 | 0.999252 | 0.7105 |
| 313.15 |  | 0.994576 | 0.997206 | 0.7117 |
| 318.15 |  | 0.992357 | 0.994986 | 0.7127 |
| 323.15 |  | 0.989972 | 0.992601 | 0.7137 |
| 328.15 |  | 0.987431 | 0.990060 | 0.7148 |
| 333.15 |  | 0.984741 | 0.987369 | 0.7161 |
| 338.15 |  | 0.981914 | 0.984526 | 0.7191 |
| 343.15 |  | 0.978949 | 0.981550 | 0.7216 |
| 348.15 |  | 0.975857 | 0.978453 | 0.7236 |
| 353.15 |  | 0.972643 | 0.975234 | 0.7255 |
| 303.15 | 20  (17.6)^a^ | 0.998500 | 1.003736 | 0.7053 |
| 308.15 |  | 0.996644 | 1.001871 | 0.7066 |
| 313.15 |  | 0.994599 | 0.999820 | 0.7077 |
| 318.15 |  | 0.992376 | 0.997594 | 0.7088 |
| 323.15 |  | 0.989997 | 0.995205 | 0.7104 |
| 328.15 |  | 0.987458 | 0.992660 | 0.7118 |
| 333.15 |  | 0.984770 | 0.989963 | 0.7134 |
| 338.15 |  | 0.981942 | 0.987107 | 0.7163 |
| 343.15 |  | 0.978977 | 0.984118 | 0.7189 |
| 348.15 |  | 0.975886 | 0.981019 | 0.7207 |
| 353.15 |  | 0.972669 | 0.977797 | 0.7224 |

^a^ concentration value determined spectroscopically taken as mass fraction [mg_protein_/g_solution_]
